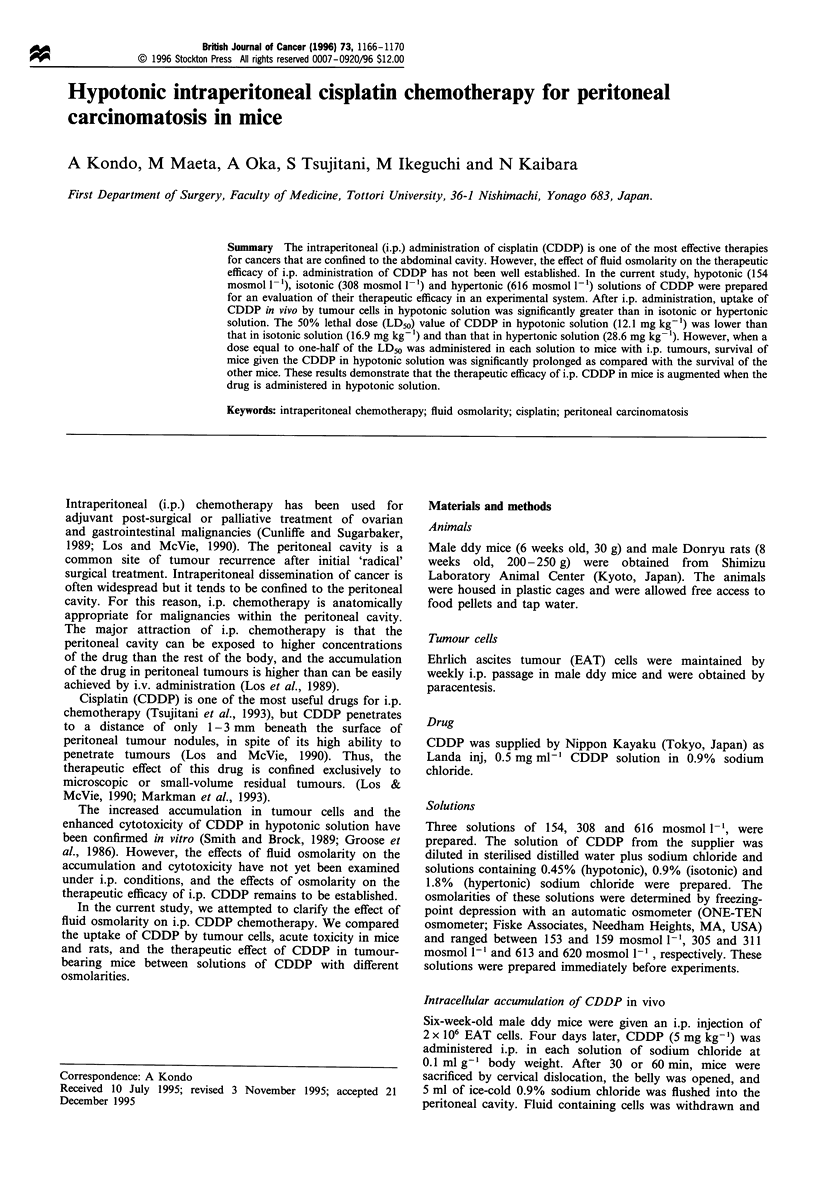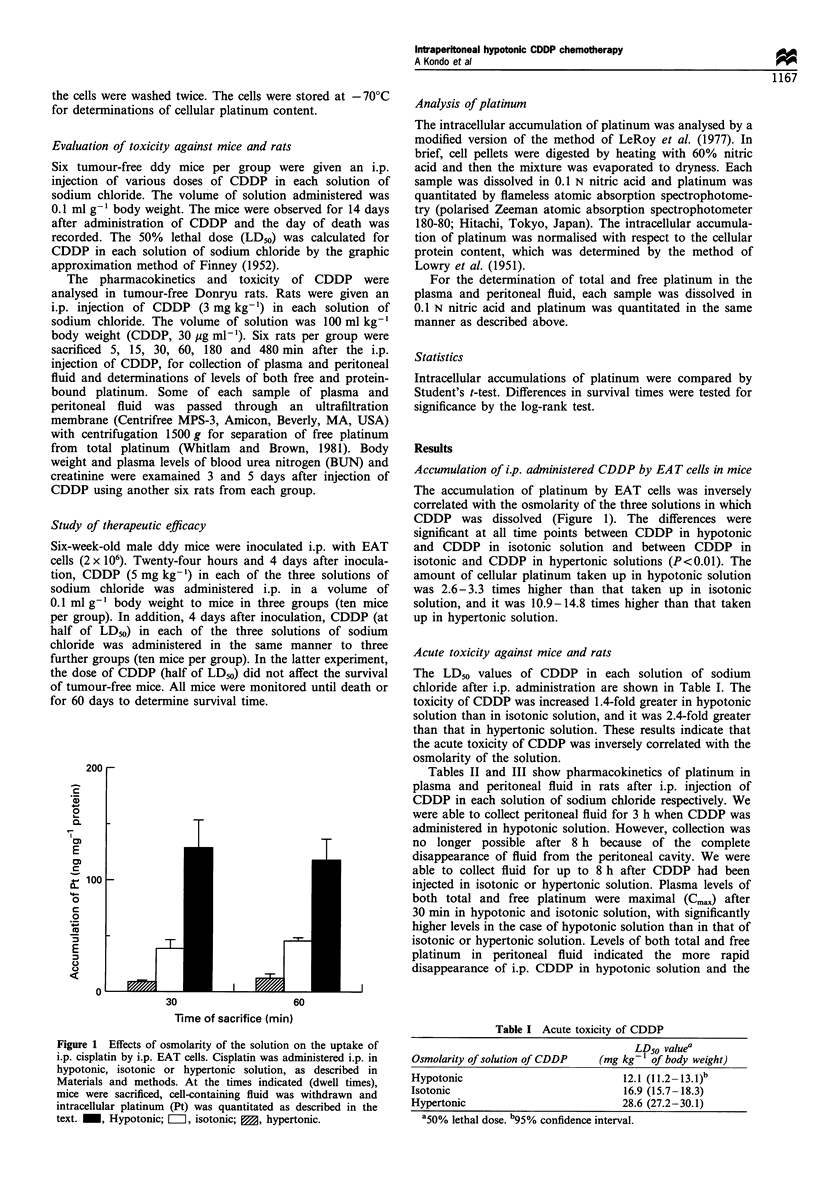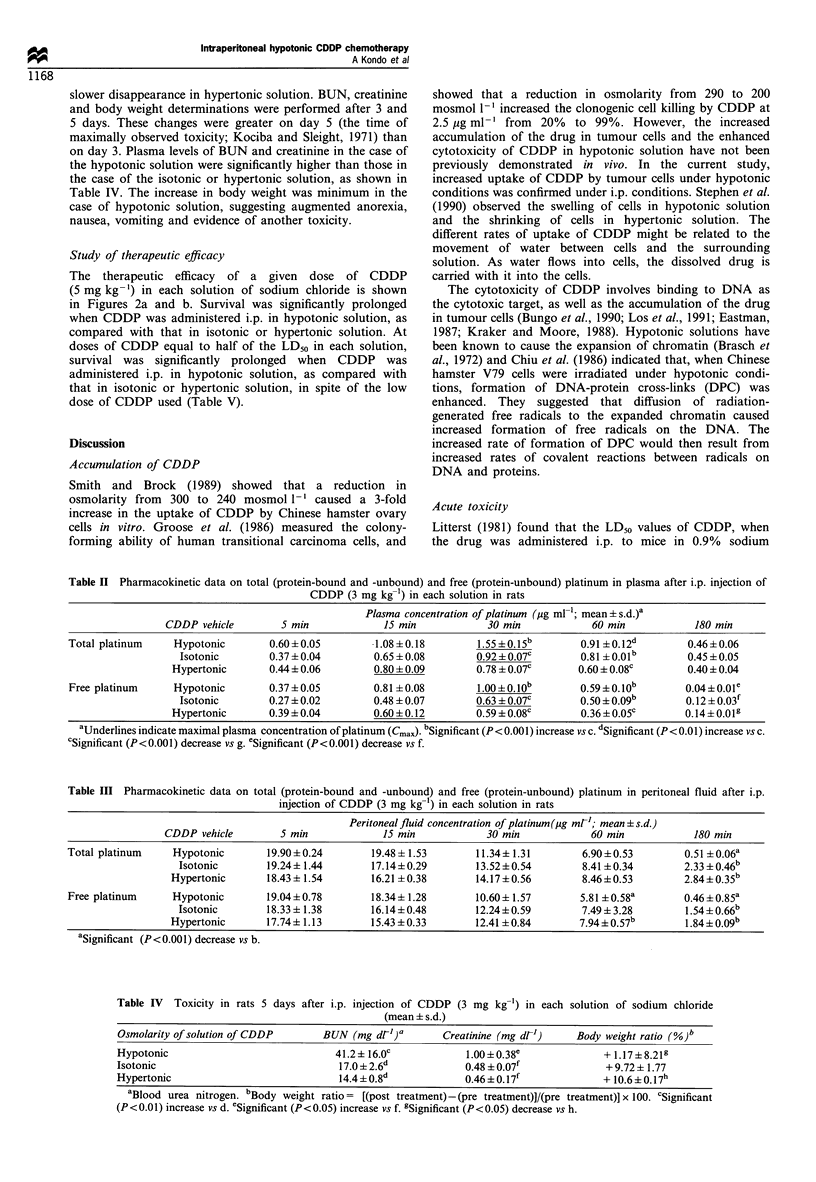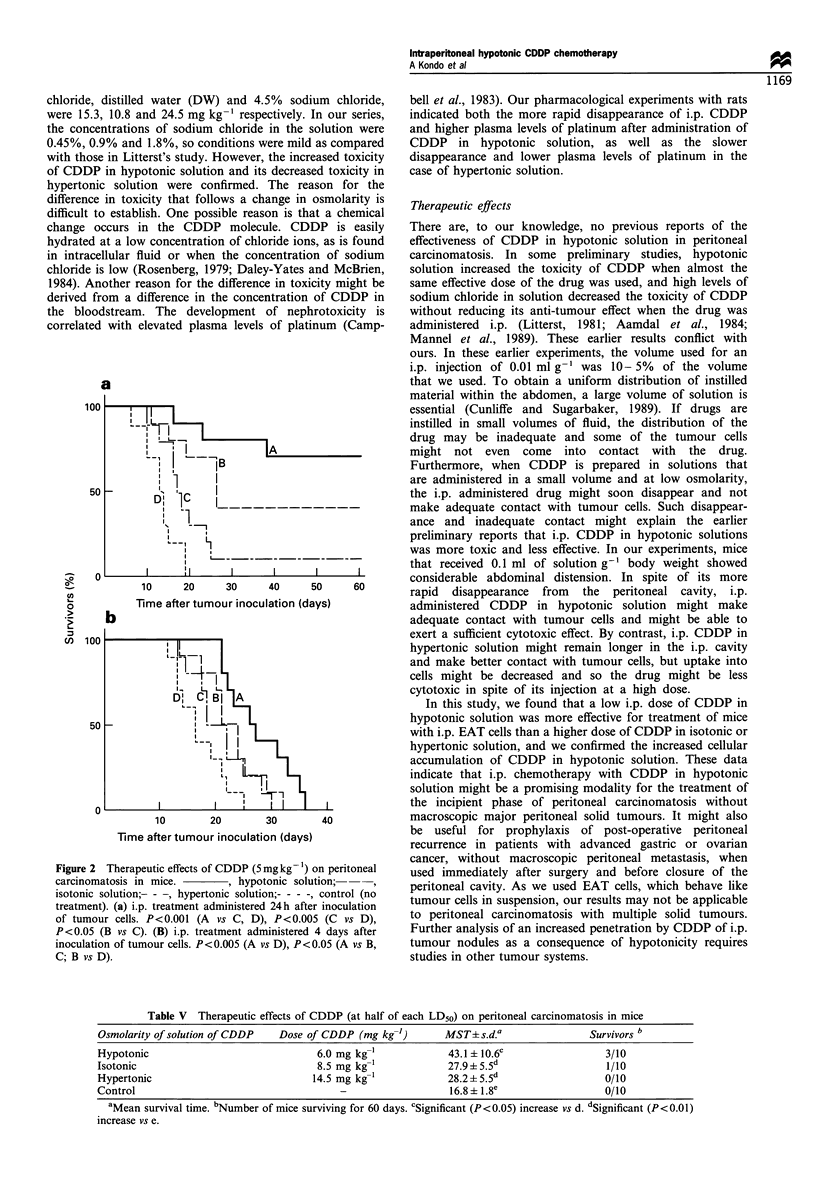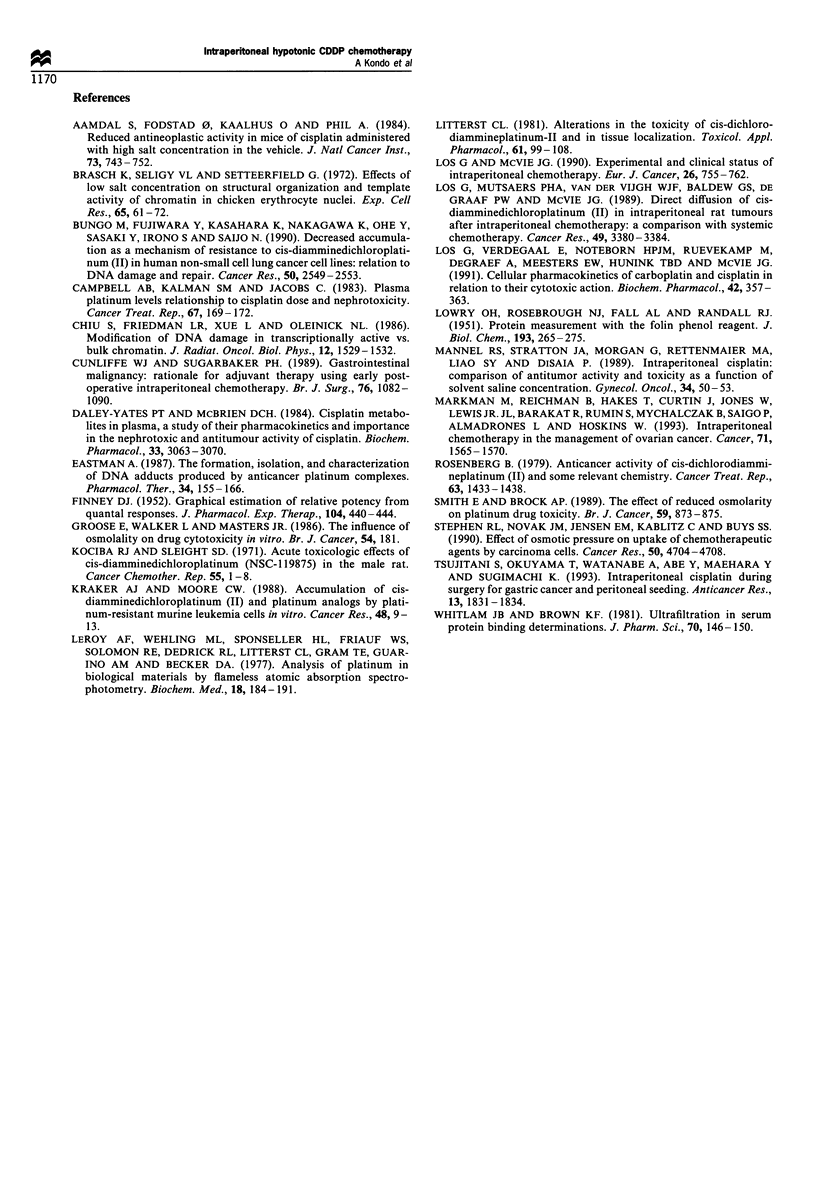# Hypotonic intraperitoneal cisplatin chemotherapy for peritoneal carcinomatosis in mice.

**DOI:** 10.1038/bjc.1996.225

**Published:** 1996-05

**Authors:** A. Kondo, M. Maeta, A. Oka, S. Tsujitani, M. Ikeguchi, N. Kaibara

**Affiliations:** First Department of Surgery, Faculty of Medicine, Tottori University, Yonago, Japan.

## Abstract

The intraperitoneal (i.p.) administration of cisplatin (CDDP) is one of the most effective therapies for cancers that are confined to the abdominal cavity. However, the effect of fluid osmolarity on the therapeutic efficacy of i.p. administration of CDDP has not been well established. In the current study, hypotonic (154 mosmol 1-1), isotonic (308 mosmol 1-1) and hypertonic (616 mosmol 1-1) solutions of CDDP were prepared for an evaluation of their therapeutic efficacy in an experimental system. After i.p. administration, uptake of CDDP in vivo by tumor cells in hypotonic solution was significantly greater than in isotonic or hypertonic solution. The 50% lethal dose (LD50) value of CDDP in hypotonic solution (12.1 mg kg(-1)) was lower than that in isotonic solution (16.9 mg kg(-1)) and than that in hypertonic solution (28.6 mg kg(-1)). However, when a dose equal to one-half of the LD50 was administered in each solution to mice with i.p. tumours, survival of mice given the CDDP in hypotonic solution was significantly prolonged as compared with the survival of the other mice. These results demonstrate that the therapeutic efficacy of i.p. CDDP in mice is augmented when the drug is administered in hypotonic solution.


British Journal of Cancer (1996) 73, 1166-1170
© 1996 Stockton Press All rights reserved 0007-0920/96 $12.00

### Hypotonic intraperitoneal cisplatin chemotherapy for peritoneal carcinomatosis in mice

A Kondo, M Maeta, A Oka, S Tsujitani, M Ikeguchi and N Kaibara

*First Department of Surgery, Faculty of Medicine, Tottori University, 36-1 Nishimachi, Yonago 683, Japan.*

**Summary** The intraperitoneal (i.p.) administration of cisplatin (CDDP) is one of the most effective therapies for cancers that are confined to the abdominal cavity. However, the effect of fluid osmolarity on the therapeutic efficacy of i.p. administration of CDDP has not been well established. In the current study, hypotonic (154 mosmol $l^{-1}$), isotonic (308 mosmol $l^{-1}$) and hypertonic (616 mosmol $l^{-1}$) solutions of CDDP were prepared for an evaluation of their therapeutic efficacy in an experimental system. After i.p. administration, uptake of CDDP *in vivo* by tumour cells in hypotonic solution was significantly greater than in isotonic or hypertonic solution. The 50% lethal dose ($LD_{50}$) value of CDDP in hypotonic solution (12.1 mg $kg^{-1}$) was lower than that in isotonic solution (16.9 mg $kg^{-1}$) and than that in hypertonic solution (28.6 mg $kg^{-1}$). However, when a dose equal to one-half of the $LD_{50}$ was administered in each solution to mice with i.p. tumours, survival of mice given the CDDP in hypotonic solution was significantly prolonged as compared with the survival of the other mice. These results demonstrate that the therapeutic efficacy of i.p. CDDP in mice is augmented when the drug is administered in hypotonic solution.

**Keywords:** intraperitoneal chemotherapy; fluid osmolarity; cisplatin; peritoneal carcinomatosis

Intraperitoneal (i.p.) chemotherapy has been used for adjuvant post-surgical or palliative treatment of ovarian and gastrointestinal malignancies (Cunliffe and Sugarbaker, 1989; Los and McVie, 1990). The peritoneal cavity is a common site of tumour recurrence after initial 'radical' surgical treatment. Intraperitoneal dissemination of cancer is often widespread but it tends to be confined to the peritoneal cavity. For this reason, i.p. chemotherapy is anatomically appropriate for malignancies within the peritoneal cavity. The major attraction of i.p. chemotherapy is that the peritoneal cavity can be exposed to higher concentrations of the drug than the rest of the body, and the accumulation of the drug in peritoneal tumours is higher than can be easily achieved by i.v. administration (Los *et al.*, 1989).

Cisplatin (CDDP) is one of the most useful drugs for i.p. chemotherapy (Tsujitani *et al.*, 1993), but CDDP penetrates to a distance of only 1-3 mm beneath the surface of peritoneal tumour nodules, in spite of its high ability to penetrate tumours (Los and McVie, 1990). Thus, the therapeutic effect of this drug is confined exclusively to microscopic or small-volume residual tumours. (Los & McVie, 1990; Markman *et al.*, 1993).

The increased accumulation in tumour cells and the enhanced cytotoxicity of CDDP in hypotonic solution have been confirmed *in vitro* (Smith and Brock, 1989; Groose *et al.*, 1986). However, the effects of fluid osmolarity on the accumulation and cytotoxicity have not yet been examined under i.p. conditions, and the effects of osmolarity on the therapeutic efficacy of i.p. CDDP remains to be established.

In the current study, we attempted to clarify the effect of fluid osmolarity on i.p. CDDP chemotherapy. We compared the uptake of CDDP by tumour cells, acute toxicity in mice and rats, and the therapeutic effect of CDDP in tumour-bearing mice between solutions of CDDP with different osmolarities.

Correspondence: A Kondo
Received 10 July 1995; revised 3 November 1995; accepted 21 December 1995

### Materials and methods

### *Animals*

Male ddy mice (6 weeks old, 30 g) and male Donryu rats (8 weeks old, 200-250 g) were obtained from Shimizu Laboratory Animal Center (Kyoto, Japan). The animals were housed in plastic cages and were allowed free access to food pellets and tap water.

### *Tumour cells*

Ehrlich ascites tumour (EAT) cells were maintained by weekly i.p. passage in male ddy mice and were obtained by paracentesis.

### *Drug*

CDDP was supplied by Nippon Kayaku (Tokyo, Japan) as Landa inj, 0.5 mg $ml^{-1}$ CDDP solution in 0.9% sodium chloride.

### *Solutions*

Three solutions of 154, 308 and 616 mosmol $l^{-1}$, were prepared. The solution of CDDP from the supplier was diluted in sterilised distilled water plus sodium chloride and solutions containing 0.45% (hypotonic), 0.9% (isotonic) and 1.8% (hypertonic) sodium chloride were prepared. The osmolarities of these solutions were determined by freezing-point depression with an automatic osmometer (ONE-TEN osmometer; Fiske Associates, Needham Heights, MA, USA) and ranged between 153 and 159 mosmol $l^{-1}$, 305 and 311 mosmol $l^{-1}$ and 613 and 620 mosmol $l^{-1}$, respectively. These solutions were prepared immediately before experiments.

### *Intracellular accumulation of CDDP* in vivo

Six-week-old male ddy mice were given an i.p. injection of $2 \times 10^6$ EAT cells. Four days later, CDDP (5 mg $kg^{-1}$) was administered i.p. in each solution of sodium chloride at 0.1 ml $g^{-1}$ body weight. After 30 or 60 min, mice were sacrificed by cervical dislocation, the belly was opened, and 5 ml of ice-cold 0.9% sodium chloride was flushed into the peritoneal cavity. Fluid containing cells was withdrawn and the cells were washed twice. The cells were stored at −70°C for determinations of cellular platinum content.

*Evaluation of toxicity against mice and rats*

Six tumour-free ddy mice per group were given an i.p. injection of various doses of CDDP in each solution of sodium chloride. The volume of solution administered was 0.1 ml $g^{-1}$ body weight. The mice were observed for 14 days after administration of CDDP and the day of death was recorded. The 50% lethal dose ($LD_{50}$) was calculated for CDDP in each solution of sodium chloride by the graphic approximation method of Finney (1952).

The pharmacokinetics and toxicity of CDDP were analysed in tumour-free Donryu rats. Rats were given an i.p. injection of CDDP (3 mg $kg^{-1}$) in each solution of sodium chloride. The volume of solution was 100 ml $kg^{-1}$ body weight (CDDP, 30 $\mu$g $ml^{-1}$). Six rats per group were sacrificed 5, 15, 30, 60, 180 and 480 min after the i.p. injection of CDDP, for collection of plasma and peritoneal fluid and determinations of levels of both free and protein-bound platinum. Some of each sample of plasma and peritoneal fluid was passed through an ultrafiltration membrane (Centrifree MPS-3, Amicon, Beverly, MA, USA) with centrifugation 1500 *g* for separation of free platinum from total platinum (Whitlam and Brown, 1981). Body weight and plasma levels of blood urea nitrogen (BUN) and creatinine were examained 3 and 5 days after injection of CDDP using another six rats from each group.

*Study of therapeutic efficacy*

Six-week-old male ddy mice were inoculated i.p. with EAT cells ($2 \times 10^6$). Twenty-four hours and 4 days after inoculation, CDDP (5 mg $kg^{-1}$) in each of the three solutions of sodium chloride was administered i.p. in a volume of 0.1 ml $g^{-1}$ body weight to mice in three groups (ten mice per group). In addition, 4 days after inoculation, CDDP (at half of $LD_{50}$) in each of the three solutions of sodium chloride was administered in the same manner to three further groups (ten mice per group). In the latter experiment, the dose of CDDP (half of $LD_{50}$) did not affect the survival of tumour-free mice. All mice were monitored until death or for 60 days to determine survival time.

**Figure 1** Effects of osmolarity of the solution on the uptake of i.p. cisplatin by i.p. EAT cells. Cisplatin was administered i.p. in hypotonic, isotonic or hypertonic solution, as described in Materials and methods. At the times indicated (dwell times), mice were sacrificed, cell-containing fluid was withdrawn and intracellular platinum (Pt) was quantitated as described in the text. ■, Hypotonic; □, isotonic; ▨, hypertonic.

*Analysis of platinum*

The intracellular accumulation of platinum was analysed by a modified version of the method of LeRoy *et al.* (1977). In brief, cell pellets were digested by heating with 60% nitric acid and then the mixture was evaporated to dryness. Each sample was dissolved in 0.1 N nitric acid and platinum was quantitated by flameless atomic absorption spectrophotometry (polarised Zeeman atomic absorption spectrophotometer 180-80; Hitachi, Tokyo, Japan). The intracellular accumulation of platinum was normalised with respect to the cellular protein content, which was determined by the method of Lowry *et al.* (1951).

For the determination of total and free platinum in the plasma and peritoneal fluid, each sample was dissolved in 0.1 N nitric acid and platinum was quantitated in the same manner as described above.

*Statistics*

Intracellular accumulations of platinum were compared by Student's *t*-test. Differences in survival times were tested for significance by the log-rank test.

### Results

*Accumulation of i.p. administered CDDP by EAT cells in mice*

The accumulation of platinum by EAT cells was inversely correlated with the osmolarity of the three solutions in which CDDP was dissolved (Figure 1). The differences were significant at all time points between CDDP in hypotonic and CDDP in isotonic solution and between CDDP in isotonic and CDDP in hypertonic solutions ($P<0.01$). The amount of cellular platinum taken up in hypotonic solution was 2.6–3.3 times higher than that taken up in isotonic solution, and it was 10.9–14.8 times higher than that taken up in hypertonic solution.

*Acute toxicity against mice and rats*

The $LD_{50}$ values of CDDP in each solution of sodium chloride after i.p. administration are shown in Table I. The toxicity of CDDP was increased 1.4-fold greater in hypotonic solution than in isotonic solution, and it was 2.4-fold greater than that in hypertonic solution. These results indicate that the acute toxicity of CDDP was inversely correlated with the osmolarity of the solution.

Tables II and III show pharmacokinetics of platinum in plasma and peritoneal fluid in rats after i.p. injection of CDDP in each solution of sodium chloride respectively. We were able to collect peritoneal fluid for 3 h when CDDP was administered in hypotonic solution. However, collection was no longer possible after 8 h because of the complete disappearance of fluid from the peritoneal cavity. We were able to collect fluid for up to 8 h after CDDP had been injected in isotonic or hypertonic solution. Plasma levels of both total and free platinum were maximal ($C_{max}$) after 30 min in hypotonic and isotonic solution, with significantly higher levels in the case of hypotonic solution than in that of isotonic or hypertonic solution. Levels of both total and free platinum in peritoneal fluid indicated the more rapid disappearance of i.p. CDDP in hypotonic solution and the

**Table I** Acute toxicity of CDDP

| *Osmolarity of solution of CDDP* | *$LD_{50}$ value[a] (mg $kg^{-1}$ of body weight)* |
|---|---|
| Hypotonic | 12.1 (11.2–13.1)[b] |
| Isotonic | 16.9 (15.7–18.3) |
| Hypertonic | 28.6 (27.2–30.1) |

[a]50% lethal dose. [b]95% confidence interval.

slower disappearance in hypertonic solution. BUN, creatinine and body weight determinations were performed after 3 and 5 days. These changes were greater on day 5 (the time of maximally observed toxicity; Kociba and Sleight, 1971) than on day 3. Plasma levels of BUN and creatinine in the case of the hypotonic solution were significantly higher than those in the case of the isotonic or hypertonic solution, as shown in Table IV. The increase in body weight was minimum in the case of hypotonic solution, suggesting augmented anorexia, nausea, vomiting and evidence of another toxicity.

### *Study of therapeutic efficacy*

The therapeutic efficacy of a given dose of CDDP (5 mg $kg^{-1}$) in each solution of sodium chloride is shown in Figures 2a and b. Survival was significantly prolonged when CDDP was administered i.p. in hypotonic solution, as compared with that in isotonic or hypertonic solution. At doses of CDDP equal to half of the $LD_{50}$ in each solution, survival was significantly prolonged when CDDP was administered i.p. in hypotonic solution, as compared with that in isotonic or hypertonic solution, in spite of the low dose of CDDP used (Table V).

### Discussion

### *Accumulation of CDDP*

Smith and Brock (1989) showed that a reduction in osmolarity from 300 to 240 mosmol $l^{-1}$ caused a 3-fold increase in the uptake of CDDP by Chinese hamster ovary cells *in vitro*. Groose *et al.* (1986) measured the colony-forming ability of human transitional carcinoma cells, and showed that a reduction in osmolarity from 290 to 200 mosmol $l^{-1}$ increased the clonogenic cell killing by CDDP at 2.5 $\mu g\ ml^{-1}$ from 20% to 99%. However, the increased accumulation of the drug in tumour cells and the enhanced cytotoxicity of CDDP in hypotonic solution have not been previously demonstrated *in vivo*. In the current study, increased uptake of CDDP by tumour cells under hypotonic conditions was confirmed under i.p. conditions. Stephen *et al.* (1990) observed the swelling of cells in hypotonic solution and the shrinking of cells in hypertonic solution. The different rates of uptake of CDDP might be related to the movement of water between cells and the surrounding solution. As water flows into cells, the dissolved drug is carried with it into the cells.

The cytotoxicity of CDDP involves binding to DNA as the cytotoxic target, as well as the accumulation of the drug in tumour cells (Bungo *et al.*, 1990; Los *et al.*, 1991; Eastman, 1987; Kraker and Moore, 1988). Hypotonic solutions have been known to cause the expansion of chromatin (Brasch *et al.*, 1972) and Chiu *et al.* (1986) indicated that, when Chinese hamster V79 cells were irradiated under hypotonic conditions, formation of DNA-protein cross-links (DPC) was enhanced. They suggested that diffusion of radiation-generated free radicals to the expanded chromatin caused increased formation of free radicals on the DNA. The increased rate of formation of DPC would then result from increased rates of covalent reactions between radicals on DNA and proteins.

### *Acute toxicity*

Litterst (1981) found that the $LD_{50}$ values of CDDP, when the drug was administered i.p. to mice in 0.9% sodium

**Table II** Pharmacokinetic data on total (protein-bound and -unbound) and free (protein-unbound) platinum in plasma after i.p. injection of CDDP (3 mg $kg^{-1}$) in each solution in rats

| | CDDP vehicle | Plasma concentration of platinum ($\mu g\ ml^{-1}$; mean ± s.d.)[a] 5 min | 15 min | 30 min | 60 min | 180 min |
|---|---|---|---|---|---|---|
| Total platinum | Hypotonic | 0.60 ± 0.05 | 1.08 ± 0.18 | 1.55 ± 0.15[b] | 0.91 ± 0.12[d] | 0.46 ± 0.06 |
| | Isotonic | 0.37 ± 0.04 | 0.65 ± 0.08 | 0.92 ± 0.07[c] | 0.81 ± 0.01[b] | 0.45 ± 0.05 |
| | Hypertonic | 0.44 ± 0.06 | 0.80 ± 0.09 | 0.78 ± 0.07[c] | 0.60 ± 0.08[c] | 0.40 ± 0.04 |
| Free platinum | Hypotonic | 0.37 ± 0.05 | 0.81 ± 0.08 | 1.00 ± 0.10[b] | 0.59 ± 0.10[b] | 0.04 ± 0.01[e] |
| | Isotonic | 0.27 ± 0.02 | 0.48 ± 0.07 | 0.63 ± 0.07[c] | 0.50 ± 0.09[b] | 0.12 ± 0.03[f] |
| | Hypertonic | 0.39 ± 0.04 | 0.60 ± 0.12 | 0.59 ± 0.08[c] | 0.36 ± 0.05[c] | 0.14 ± 0.01[g] |

[a]Underlines indicate maximal plasma concentration of platinum ($C_{max}$). [b]Significant ($P < 0.001$) increase *vs* c. [d]Significant ($P < 0.01$) increase *vs* c. [c]Significant ($P < 0.001$) decrease *vs* g. [e]Significant ($P < 0.001$) decrease *vs* f.

**Table III** Pharmacokinetic data on total (protein-bound and -unbound) and free (protein-unbound) platinum in peritoneal fluid after i.p. injection of CDDP (3 mg $kg^{-1}$) in each solution in rats

| | CDDP vehicle | Peritoneal fluid concentration of platinum ($\mu g\ ml^{-1}$; mean ± s.d.) 5 min | 15 min | 30 min | 60 min | 180 min |
|---|---|---|---|---|---|---|
| Total platinum | Hypotonic | 19.90 ± 0.24 | 19.48 ± 1.53 | 11.34 ± 1.31 | 6.90 ± 0.53 | 0.51 ± 0.06[a] |
| | Isotonic | 19.24 ± 1.44 | 17.14 ± 0.29 | 13.52 ± 0.54 | 8.41 ± 0.34 | 2.33 ± 0.46[b] |
| | Hypertonic | 18.43 ± 1.54 | 16.21 ± 0.38 | 14.17 ± 0.56 | 8.46 ± 0.53 | 2.84 ± 0.35[b] |
| Free platinum | Hypotonic | 19.04 ± 0.78 | 18.34 ± 1.28 | 10.60 ± 1.57 | 5.81 ± 0.58[a] | 0.46 ± 0.85[a] |
| | Isotonic | 18.33 ± 1.38 | 16.14 ± 0.48 | 12.24 ± 0.59 | 7.49 ± 3.28 | 1.54 ± 0.66[b] |
| | Hypertonic | 17.74 ± 1.13 | 15.43 ± 0.33 | 12.41 ± 0.84 | 7.94 ± 0.57[b] | 1.84 ± 0.09[b] |

[a]Significant ($P < 0.001$) decrease *vs* b.

**Table IV** Toxicity in rats 5 days after i.p. injection of CDDP (3 mg $kg^{-1}$) in each solution of sodium chloride (mean ± s.d.)

| Osmolarity of solution of CDDP | BUN (mg $dl^{-1}$)[a] | Creatinine (mg $dl^{-1}$) | Body weight ratio (%)[b] |
|---|---|---|---|
| Hypotonic | 41.2 ± 16.0[c] | 1.00 ± 0.38[e] | + 1.17 ± 8.21[g] |
| Isotonic | 17.0 ± 2.6[d] | 0.48 ± 0.07[f] | + 9.72 ± 1.77 |
| Hypertonic | 14.4 ± 0.8[d] | 0.46 ± 0.17[f] | + 10.6 ± 0.17[h] |

[a]Blood urea nitrogen. [b]Body weight ratio = [(post treatment) − (pre treatment)]/(pre treatment)] × 100. [c]Significant ($P < 0.01$) increase *vs* d. [e]Significant ($P < 0.05$) increase *vs* f. [g]Significant ($P < 0.05$) decrease *vs* h.

chloride, distilled water (DW) and 4.5% sodium chloride, were 15.3, 10.8 and 24.5 mg $kg^{-1}$ respectively. In our series, the concentrations of sodium chloride in the solution were 0.45%, 0.9% and 1.8%, so conditions were mild as compared with those in Litterst's study. However, the increased toxicity of CDDP in hypotonic solution and its decreased toxicity in hypertonic solution were confirmed. The reason for the difference in toxicity that follows a change in osmolarity is difficult to establish. One possible reason is that a chemical change occurs in the CDDP molecule. CDDP is easily hydrated at a low concentration of chloride ions, as is found in intracellular fluid or when the concentration of sodium chloride is low (Rosenberg, 1979; Daley-Yates and McBrien, 1984). Another reason for the difference in toxicity might be derived from a difference in the concentration of CDDP in the bloodstream. The development of nephrotoxicity is correlated with elevated plasma levels of platinum (Campbell *et al.*, 1983). Our pharmacological experiments with rats indicated both the more rapid disappearance of i.p. CDDP and higher plasma levels of platinum after administration of CDDP in hypotonic solution, as well as the slower disappearance and lower plasma levels of platinum in the case of hypertonic solution.

**Figure 2** Therapeutic effects of CDDP (5 mg $kg^{-1}$) on peritoneal carcinomatosis in mice. ————, hypotonic solution;———, isotonic solution;– – –, hypertonic solution;- - - -, control (no treatment). (**a**) i.p. treatment administered 24 h after inoculation of tumour cells. $P<0.001$ (A *vs* C, D), $P<0.005$ (C *vs* D), $P<0.05$ (B *vs* C). (**B**) i.p. treatment administered 4 days after inoculation of tumour cells. $P<0.005$ (A *vs* D), $P<0.05$ (A *vs* B, C; B *vs* D).

*Therapeutic effects*

There are, to our knowledge, no previous reports of the effectiveness of CDDP in hypotonic solution in peritoneal carcinomatosis. In some preliminary studies, hypotonic solution increased the toxicity of CDDP when almost the same effective dose of the drug was used, and high levels of sodium chloride in solution decreased the toxicity of CDDP without reducing its anti-tumour effect when the drug was administered i.p. (Litterst, 1981; Aamdal *et al.*, 1984; Mannel *et al.*, 1989). These earlier results conflict with ours. In these earlier experiments, the volume used for an i.p. injection of 0.01 ml $g^{-1}$ was 10–5% of the volume that we used. To obtain a uniform distribution of instilled material within the abdomen, a large volume of solution is essential (Cunliffe and Sugarbaker, 1989). If drugs are instilled in small volumes of fluid, the distribution of the drug may be inadequate and some of the tumour cells might not even come into contact with the drug. Furthermore, when CDDP is prepared in solutions that are administered in a small volume and at low osmolarity, the i.p. administered drug might soon disappear and not make adequate contact with tumour cells. Such disappearance and inadequate contact might explain the earlier preliminary reports that i.p. CDDP in hypotonic solutions was more toxic and less effective. In our experiments, mice that received 0.1 ml of solution $g^{-1}$ body weight showed considerable abdominal distension. In spite of its more rapid disappearance from the peritoneal cavity, i.p. administered CDDP in hypotonic solution might make adequate contact with tumour cells and might be able to exert a sufficient cytotoxic effect. By contrast, i.p. CDDP in hypertonic solution might remain longer in the i.p. cavity and make better contact with tumour cells, but uptake into cells might be decreased and so the drug might be less cytotoxic in spite of its injection at a high dose.

In this study, we found that a low i.p. dose of CDDP in hypotonic solution was more effective for treatment of mice with i.p. EAT cells than a higher dose of CDDP in isotonic or hypertonic solution, and we confirmed the increased cellular accumulation of CDDP in hypotonic solution. These data indicate that i.p. chemotherapy with CDDP in hypotonic solution might be a promising modality for the treatment of the incipient phase of peritoneal carcinomatosis without macroscopic major peritoneal solid tumours. It might also be useful for prophylaxis of post-operative peritoneal recurrence in patients with advanced gastric or ovarian cancer, without macroscopic peritoneal metastasis, when used immediately after surgery and before closure of the peritoneal cavity. As we used EAT cells, which behave like tumour cells in suspension, our results may not be applicable to peritoneal carcinomatosis with multiple solid tumours. Further analysis of an increased penetration by CDDP of i.p. tumour nodules as a consequence of hypotonicity requires studies in other tumour systems.

**Table V** Therapeutic effects of CDDP (at half of each $LD_{50}$) on peritoneal carcinomatosis in mice

| *Osmolarity of solution of CDDP* | *Dose of CDDP (mg $kg^{-1}$)* | *MST ± s.d.*[a] | *Survivors*[b] |
|---|---|---|---|
| Hypotonic | 6.0 mg $kg^{-1}$ | 43.1 ± 10.6[c] | 3/10 |
| Isotonic | 8.5 mg $kg^{-1}$ | 27.9 ± 5.5[d] | 1/10 |
| Hypertonic | 14.5 mg $kg^{-1}$ | 28.2 ± 5.5[d] | 0/10 |
| Control | – | 16.8 ± 1.8[e] | 0/10 |

[a]Mean survival time. [b]Number of mice surviving for 60 days. [c]Significant ($P<0.05$) increase *vs* d. [d]Significant ($P<0.01$) increase *vs* e.

### References

AAMDAL S, FODSTAD Ø, KAALHUS O AND PHIL A. (1984). Reduced antineoplastic activity in mice of cisplatin administered with high salt concentration in the vehicle. *J. Natl Cancer Inst.*, **73**, 743–752.

BRASCH K, SELIGY VL AND SETTEERFIELD G. (1972). Effects of low salt concentration on structural organization and template activity of chromatin in chicken erythrocyte nuclei. *Exp. Cell Res.*, **65**, 61–72.

BUNGO M, FUJIWARA Y, KASAHARA K, NAKAGAWA K, OHE Y, SASAKI Y, IRONO S AND SAIJO N. (1990). Decreased accumulation as a mechanism of resistance to cis-diamminedichloroplatinum (II) in human non-small cell lung cancer cell lines: relation to DNA damage and repair. *Cancer Res.*, **50**, 2549–2553.

CAMPBELL AB, KALMAN SM AND JACOBS C. (1983). Plasma platinum levels relationship to cisplatin dose and nephrotoxicity. *Cancer Treat. Rep.*, **67**, 169–172.

CHIU S, FRIEDMAN LR, XUE L AND OLEINICK NL. (1986). Modification of DNA damage in transcriptionally active vs. bulk chromatin. *J. Radiat. Oncol. Biol. Phys.*, **12**, 1529–1532.

CUNLIFFE WJ AND SUGARBAKER PH. (1989). Gastrointestinal malignancy: rationale for adjuvant therapy using early post-operative intraperitoneal chemotherapy. *Br. J. Surg.*, **76**, 1082–1090.

DALEY-YATES PT AND MCBRIEN DCH. (1984). Cisplatin metabolites in plasma, a study of their pharmacokinetics and importance in the nephrotoxic and antitumour activity of cisplatin. *Biochem. Pharmacol.*, **33**, 3063–3070.

EASTMAN A. (1987). The formation, isolation, and characterization of DNA adducts produced by anticancer platinum complexes. *Pharmacol. Ther.*, **34**, 155–166.

FINNEY DJ. (1952). Graphical estimation of relative potency from quantal responses. *J. Pharmacol. Exp. Therap.*, **104**, 440–444.

GROOSE E, WALKER L AND MASTERS JR. (1986). The influence of osmolality on drug cytotoxicity *in vitro*. *Br. J. Cancer*, **54**, 181.

KOCIBA RJ AND SLEIGHT SD. (1971). Acute toxicologic effects of cis-diamminedichloroplatinum (NSC-119875) in the male rat. *Cancer Chemother. Rep.* **55**, 1–8.

KRAKER AJ AND MOORE CW. (1988). Accumulation of cis-diamminedichloroplatinum (II) and platinum analogs by platinum-resistant murine leukemia cells *in vitro*. *Cancer Res.*, **48**, 9–13.

LEROY AF, WEHLING ML, SPONSELLER HL, FRIAUF WS, SOLOMON RE, DEDRICK RL, LITTERST CL, GRAM TE, GUARINO AM AND BECKER DA. (1977). Analysis of platinum in biological materials by flameless atomic absorption spectrophotometry. *Biochem. Med.*, **18**, 184–191.

LITTERST CL. (1981). Alterations in the toxicity of cis-dichlorodiammineplatinum-II and in tissue localization. *Toxicol. Appl. Pharmacol.*, **61**, 99–108.

LOS G AND MCVIE JG. (1990). Experimental and clinical status of intraperitoneal chemotherapy. *Eur. J. Cancer*, **26**, 755–762.

LOS G, MUTSAERS PHA, VAN DER VIJGH WJF, BALDEW GS, DE GRAAF PW AND MCVIE JG. (1989). Direct diffusion of cis-diamminedichloroplatinum (II) in intraperitoneal rat tumours after intraperitoneal chemotherapy: a comparison with systemic chemotherapy. *Cancer Res.*, **49**, 3380–3384.

LOS G, VERDEGAAL E, NOTEBORN HPJM, RUEVEKAMP M, DEGRAEF A, MEESTERS EW, HUNINK TBD AND MCVIE JG. (1991). Cellular pharmacokinetics of carboplatin and cisplatin in relation to their cytotoxic action. *Biochem. Pharmacol.*, **42**, 357–363.

LOWRY OH, ROSEBROUGH NJ, FALL AL AND RANDALL RJ. (1951). Protein measurement with the folin phenol reagent. *J. Biol. Chem.*, **193**, 265–275.

MANNEL RS, STRATTON JA, MORGAN G, RETTENMAIER MA, LIAO SY AND DISAIA P. (1989). Intraperitoneal cisplatin: comparison of antitumor activity and toxicity as a function of solvent saline concentration. *Gynecol. Oncol.*, **34**, 50–53.

MARKMAN M, REICHMAN B, HAKES T, CURTIN J, JONES W, LEWIS JR. JL, BARAKAT R, RUMIN S, MYCHALCZAK B, SAIGO P, ALMADRONES L AND HOSKINS W. (1993). Intraperitoneal chemotherapy in the management of ovarian cancer. *Cancer*, **71**, 1565–1570.

ROSENBERG B. (1979). Anticancer activity of cis-dichlorodiammineplatinum (II) and some relevant chemistry. *Cancer Treat. Rep.*, **63**, 1433–1438.

SMITH E AND BROCK AP. (1989). The effect of reduced osmolarity on platinum drug toxicity. *Br. J. Cancer*, **59**, 873–875.

STEPHEN RL, NOVAK JM, JENSEN EM, KABLITZ C AND BUYS SS. (1990). Effect of osmotic pressure on uptake of chemotherapeutic agents by carcinoma cells. *Cancer Res.*, **50**, 4704–4708.

TSUJITANI S, OKUYAMA T, WATANABE A, ABE Y, MAEHARA Y AND SUGIMACHI K. (1993). Intraperitoneal cisplatin during surgery for gastric cancer and peritoneal seeding. *Anticancer Res.*, **13**, 1831–1834.

WHITLAM JB AND BROWN KF. (1981). Ultrafiltration in serum protein binding determinations. *J. Pharm. Sci.*, **70**, 146–150.